# Association of glucose and blood pressure variability on oxidative stress in patients with type 2 diabetes mellitus and hypertension: a cross-sectional study

**DOI:** 10.1186/s13098-019-0425-y

**Published:** 2019-04-11

**Authors:** Makoto Ohara, Yo Kohata, Hiroe Nagaike, Masakazu Koshibu, Hiroya Gima, Munenori Hiromura, Takeshi Yamamoto, Yusaku Mori, Toshiyuki Hayashi, Tomoyasu Fukui, Tsutomu Hirano

**Affiliations:** Division of Diabetes, Metabolism, and Endocrinology, Department of Medicine, Showa University School of Medicine, 1-5-8 Hatanodai, Shinagawa-ku, Tokyo, 142-8666 Japan

**Keywords:** Glucose variability, Blood pressure variability, Continuous glucose monitoring, Ambulatory blood pressure monitoring, Oxidative stress, Type 2 diabetes mellitus

## Abstract

**Background:**

The present study evaluated the effects of glucose and blood pressure (BP) variability on oxidative stress in patients with type 2 diabetes mellitus (T2DM) and hypertension.

**Methods:**

A total of 60 inpatients with T2DM underwent continuous glucose monitoring (CGM) and ambulatory BP monitoring (ABPM). Oxidative stress was estimated using the diacron-reactive oxygen metabolites (d-ROMs) test. Glucose variability, mean glucose level, percentage coefficient of variation for glucose, mean amplitude of glycemic excursions (MAGE), and area under the postprandial plasma glucose curve were determined through CGM. BP variability was assessed by measuring average BP, standard deviation (SD) of systolic and diastolic BP, and coefficient of variation (CV) of systolic and diastolic BP during daytime and nighttime ABPM.

**Results:**

Participants had a mean age of 64.5 ± 13.3 years with the duration of the disease 13.9 ± 12.4 years and HbA1c of 8.5 ± 1.2%. Univariate analysis showed that MAGE, nighttime SDs of systolic and diastolic BP, and nighttime CV of systolic BP were significantly correlated with d-ROMs. Further, stepwise multiple regression analysis identified MAGE, nighttime SD and CV of diastolic BP, estimated glomerular filtration rate, and smoking as independent contributors to d-ROMs.

**Conclusions:**

Oxidative stress was associated with daily glucose and nighttime diastolic BP variability in patients with T2DM and hypertension.

*Trial registration* UMIN Clinical Trial Registry UMIN000035615, Registered January 22, 2019—retrospectively registered

**Electronic supplementary material:**

The online version of this article (10.1186/s13098-019-0425-y) contains supplementary material, which is available to authorized users.

## Background

The coexistence of diabetes and hypertension, which is considered as highly likely, has been associated with increased risk for death, cardiovascular events, and progression of microvascular complications, such as nephropathy and retinopathy [1, 2]. In some studies, intensified intervention aimed at multiple risk factors has been shown to have beneficial effects with respect to macro and microvascular complications in patients with type 2 diabetes mellitus (T2DM) [3, 4]. In addition to chronic hyperglycemia and hypertension, recent studies have reported that not only visit-to-visit glucose variability but also visit-to-visit blood pressure (BP) variability is related to macro and microvascular complications in patients with T2DM [5, 6].

Oxidative stress plays an important role in the development and progression of diabetic complications [7]. Hyperglycemic damage results from reactive oxygen species-induced activation of polyol, hexosamine, protein kinase C, and the advanced glycation end-product pathway [8]. Given that atherosclerosis can result from glucose variability-induced endothelial dysfunction through oxidative stress, the activation of oxidative stress could be a risk factor for diabetic complications [9]. Hypertension also induces endothelial dysfunction through oxidative stress [10]. Accordingly, angiotensin II has been reported to stimulate the production of reactive oxygen species, such as superoxide, through the activation of membrane-bound NADH or NADPH oxidase in hypertension [11, 12]. In addition, nitric oxide inactivation by oxygen free radicals contributes to endothelial dysfunction in essential hypertension [13].

Short- and long-term variability has been observed for glucose and BP levels. Given the advances in medical technology, such as continuous glucose monitoring (CGM) and 24 h ambulatory blood pressure monitoring (ABPM), short-term glucose and BP variability can be detected in greater detail. Although long-term glucose variability has been associated with oxidative stress in patients with T2DM [14], cross-sectional and interventional studies have also found a correlation between short-term glucose variability and oxidative stress in patients with T2DM [15–17]. However, only a few reports have investigated the association between oxidative stress and BP variability in patients with T2DM and hypertension.

Therefore, the present study aimed to determine whether glucose and BP variability measured using CGM and ABPM, respectively, was associated with diacron-reactive oxygen metabolites (d-ROMs), a surrogate marker of oxidative stress in patients with T2DM [16, 17].

## Methods

### Study subjects

We performed a cross-sectional study, including 60 inpatients with T2DM and hypertension, who were treated at Showa University Hospital from June 2015 to January 2018. Reasons for hospital admission were to achieve glycemic control because of poor control or to evaluate glucose and BP variabilities. The inclusion criteria were as follows: a diagnosis of T2DM and hypertension, age > 20 years, and stable diabetes and hypertension treatment for ≥ 3 months before study. T2DM was defined according to the Japan Diabetes Society [18]. Hypertension was defined as a systolic blood pressure (SBP) ≥ 140 mmHg and/or diastolic blood pressure (DBP) ≥ 90 mmHg on at least two occasions according to current guidelines [19] or a previous diagnosis of hypertension with antihypertensive medication. The exclusion criteria were as follows: use of steroids or anti-inflammatory drugs, diabetic ketosis and coma within 3 months before the study, severe infection, severe trauma, malignancy, an estimated glomerular filtration rate (eGFR) of < 30 mL/min/1.73 m^2^ according to the Cockcroft–Gault formula, pre- and post-surgery, severe liver dysfunction, pregnancy, and secondary hypertension.

### Study design

**Fig. 1:**
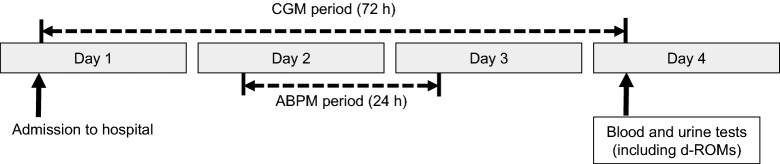
Study protocol

The study protocol is summarized in Fig. 1. This was a cross-sectional analysis of patients with T2DM who underwent a 72 h period of CGM and a 24 h period of ABPM. Clinical and laboratory parameters, including body mass index (BMI), fasting plasma glucose (FPG), HbA1c, eGFR, low-density lipoprotein cholesterol (LDL-C), high-density lipoprotein cholesterol (HDL-C), and triglycerides (TG), were measured before breakfast on day 4. Plasma oxidant capacity against *N*,*N*-diethyl paraphenylenediamine was also measured using the d-ROMs test on day 4. Clinical data (age, sex, smoking status, duration of diabetes, diabetes therapy, and antihypertensive and lipid-lowering drugs) were retrieved from medicals records. Parameters of glucose variability, such as mean glucose level (MGL), percentage coefficient of variation for glucose (%CV), mean amplitude of glycemic excursions (MAGE), and area under the postprandial plasma glucose curve (AUC_PP_), were measured on days 2 and 3. Parameters of BP variability, such as average (AV) BP, standard deviation (SD) of BP, and coefficient of variation for blood pressure (CV) were measured for 24 h beginning on day 2. All patients continued their original treatment during CGM and ABPM. The study protocol was approved by the Ethics Committee of the Showa University School of Medicine. Informed consent was obtained from all subjects after receiving a clear explanation of the study protocol. The study was designed in compliance with the Declaration of Helsinki. The present study was registered under the UMIN protocol registration system (ID: UMIN000035615).

### Procedures and measurements

Venous blood samples were drawn for laboratory measurements on day 4 before breakfast. All patients received a weight-maintaining diet (25–30 kcal/kg ideal body weight) with salt restriction (< 6 g/day). The CGM sensor (ipro2; Medtronic MiniMed, Northridge, CA, USA) was subcutaneously inserted on day 1 and removed on day 4. Using CGM data, glucose variability was calculated only on days 2 and 3 to avoid bias. The MGL was measured from the date recorded on CGM and adjusted for self-monitored blood glucose. %CV was calculated using the coefficient of variation obtained by dividing the SD by the MGL and multiplying by 100. The MAGE was calculated to assess glucose variability [20]. The AUC_PP_ was calculated using the incremental areas above preprandial glucose values 4 h after each meal [15]. After attaching the ABPM device (RAC-3502; Nihon Kohden Co., Ltd., Tokyo, Japan) on day 2, BP was measured in the left upper extremity using the oscillometric method and pulse rate at 30-min intervals for 24 h. Daytime and nighttime were defined on the basis of patients’ written diaries recorded during ABPM. BP variability was estimated using the SD and CV of SBP and DBP during the daytime and nighttime. AV SBP and DBP during the daytime and nighttime were also determined. Circadian rhythm of BP (%) was calculated as (awake SBP − asleep SPB)/awake SBP and subsequently classified as follows: dipper (fall ≥ 10%), non-dipper (fall ≥ 0% but < 10%), riser (fall < 0%), and extreme-dipper (fall ≥ 20%) [21]. The ratio of low-to-high-frequency power (LF/HF ratio) was used as a measure of sympathetic and parasympathetic activity and measured using a Holter electrocardiogram (RAC 3502; Nihon Kohden Co., Ltd., Tokyo, Japan) for 24 h on day 2. Further, coefficient of variation in R–R intervals (CVR–R) was used as a measure of sympathetic and parasympathetic activities, and it was measured using a noninvasive automatic waveform analyzer (Nihon Kohden Co., Ltd., Tokyo, Japan) on day 4.

### Laboratory measurements

Oxidative stress was measured using a d-ROMs test [22] and dedicated photometer (F.R.E.E. System, imported by LTD Tokyo from Diacron International s.r.l. Grosseto, Italy) as previously reported [23]. In accordance with the Wismerll kinetic procedure, the change in absorbance per minute was expressed as arbitrary units after correction (U.CARR, where 1 U.CARR = the oxidant capacity of a 0.08 mg/dL H_2_O_2_ solution; normal range = 250–300 U.CARR). Intra- and inter-assay coefficients of variation were 2.1% and 3.1%, respectively. Serum total cholesterol, LDL-C, HDL-C, TG, and creatinine levels were also measured using an automated analyzer (BM6070, Japan Electron Optics Laboratory, Tokyo, Japan). Plasma glucose was measured using the glucose oxidase method, whereas HbA1c was measured using high-performance liquid chromatography [24].

### Statistical analysis

Pearson’s correlation coefficient was used for univariate analysis. The Bonferroni correction was used to compare d-ROMs between four groups in low glucose variability and nighttime BP variability, high glucose variability, high BP variability, high glucose variability, and BP variability. Multiple stepwise regression analysis was then performed with d-ROMs as the dependent variable. Independent variables included sex (female), age, duration of diabetes, BMI, smoking status (current), use of insulin, glucose-like peptide-1 receptor agonists, dipeptidyl peptidase 4 inhibitors, sulfonylureas, α-glucosidase inhibitors, metformin, thiazolidine, sodium glucose cotransporter 2 inhibitors, statins, angiotensin II receptor blockers, calcium channel blockers, diuretics, α and β blockers, eGFR, FPG, MGL, HbA1c, %CV, MAGE, AUC_PP_, circadian rhythm pattern of BP, daytime AV of SBP, daytime SD of SBP, daytime CV of SBP, daytime AV of DBP, daytime SD of DBP, daytime CV of DBP, nighttime AV of SBP, nighttime SD of SBP, nighttime CV of SBP, nighttime AV of DBP, nighttime SD of DBP, nighttime CV of DBP, HDL-C, LDL-C, and TG. Analyses were performed using IBM SPSS, Version 22, for Windows (IBM Corp, Armonk, NY, USA) with a p value of < 0.05 indicating statistical significance. Data were expressed as mean ± SD.

## Results

### Clinical characteristics

The clinical and laboratory characteristics of the 60 participants are shown in Table 1. Participants had a mean age of 64.5 ± 13.3 years, mean diabetes duration of 13.9 ± 12.4 years, and a mean HbA1c level of 8.5 ± 1.2%. The study group included more men (*n* = 34) than women (*n* = 26), with participants being slightly overweight (BMI = 27.6 ± 4.5). At baseline, 35% of the patients were on metformin, 40% on sulfonylureas, 41.7% on dipeptidyl peptidase 4 inhibitors, 43.3% on angiotensin 2 receptor blockers, and 48.3% on calcium channel blockers.

**Table 1:** Baseline clinical characteristics of subjects

Clinical characteristics	Mean ± SD, n (%)
Age (years)	64.5 ± 13.3
Sex (male)	34 (56.7)
Body mass index (kg/m^2^)	27.6 ± 4.5
Smoking (%)	12 (20.0)
Drinking (%)	17 (28.3)
Duration of diabetes (years)	13.9 ± 12.4
Dyslipidemia	48 (80.0)
Low-density lipoprotein cholesterol (mg/dL)	108.3 ± 36.4
High-density lipoprotein cholesterol (mg/dL)	44.2 ± 12.0
Triglycerides (mg/dL)	152.3 ± 74.1
Estimated glomerular filtration rate (mL/min/1.73 m^2^)	75.0 ± 21.3
HbA1c (%)	8.5 ± 1.2
Mean glucose level (mg/dL)	182.2 ± 36.7
Markers of glucose variability	
%CV	23.7 ± 5.7
MAGE (mg/dL)	125.9 ± 35.2
Fasting plasma glucose state (mg/dL)	141.9 ± 33.9
AUC_PP_ (mg/dL/h)	470.6 ± 291.1
Markers of BP variability	
Daytime average of SBP (mmHg)	129.9 ± 14.7
Daytime SD of SBP (mmHg)	15.0 ± 4.5
Daytime CV of SBP (mmHg)	11.6 ± 3.5
Daytime average of DBP (mmHg)	76.6 ± 10.8
Daytime SD of DBP (mmHg)	9.9 ± 3.1
Daytime CV of DBP (mmHg)	13.0 ± 4.1
Nighttime average of SBP (mmHg)	122.2 ± 17.8
Nighttime SD of SBP (mmHg)	13.4 ± 3.9
Nighttime CV of SBP (mmHg)	11.1 ± 3.3
Nighttime average of DBP (mmHg)	70.9 ± 12.6
Nighttime SD of DBP (mmHg)	8.3 ± 2.6
Nighttime CV of DBP (mmHg)	12.0 ± 4.2
Dipper/non-dipper/riser/extreme-dipper	16/27/16/1
LF/HF ratio	3.7 ± 2.3
CVR–R (%)	2.9 ± 2.1
d-ROMs (U.CARR)	356.1 ± 66.7
Macroangiopathy	14 (23.3)
Nephropathy	25 (41.7)
Neuropathy	31 (51.7)
Retinopathy	21 (35.0)
Diabetes therapy	
Diet alone	16 (26.7)
Metformin	21 (35.0)
Sulfonylurea	24 (40.0)
Glinide	0 (0.0)
α-Glucosidase inhibitor	11 (18.3)
Thiazolidine	11 (18.3)
Dipeptidyl peptidase 4 inhibitor	25 (41.7)
Sodium glucose cotransporter 2 inhibitors	6 (10.0)
Glucose-like peptide 1 receptor agonist	7 (11.7)
Insulin	14 (23.3)
Antihypertensive drugs	
Diet alone	23 (38.3)
Angiotensin II receptor blocker	26 (43.3)
Calcium channel blocker	29 (48.3)
Diuretic	5 (8.3)
α Blocker	1 (1.7)
β Blocker	4 (6.7)
Other treatments	
Lipid-lowering drugs (statins)	33 (55.0)

Data are mean ± SD, n (%)

1 U.CARR (arbitrary unit) = the oxidant capacity of a 0.08 mg/dL H_2_O_2_ solution

*HbA1c* hemoglobin A1c, *%CV* percentage coefficient of variation for glucose, *MAGE* mean amplitude of glycemic excursions, *AUC*_*PP*_ area under the postprandial plasma glucose curve, *CV* coefficient of variation, *SD* standard deviation, *SBP* systolic blood pressure, *DBP* diastolic blood pressure, *LF/HF* low frequency power/high-frequency power, *CVR–R* coefficient of variation in the R–R intervals, *d-ROMs* diacron-reactive oxygen metabolites

### Relationship between d-ROMs, markers of diabetes and BP control, and non-glycemic clinical and laboratory variables

Table 2 shows the correlation between d-ROMs and markers of diabetic control. Significant correlations were observed between d-ROMs and %CV (r = 0.329; p = 0.010), MAGE (r = 0.448; p < 0.001), and AUC_PP_ (r = 0.307; p = 0.017). However, no significant correlation was observed between d-ROMs and FPG, MGL, HbA1c, and non-glycemic variables.

**Table 2:** Correlations between d-ROMs and markers of diabetic control and non-glycemic metabolic variables

	FPG	MGL	HbA1c	%CV	MAGE	AUC_PP_	HDL-C	LDL-C	TG
MGL	0.630**								
HbA1c	0.709**	0.659**							
%CV	− 0.252	− 0.155	− 0.100						
MAGE	0.300*	0.567**	0.334**	0.677**					
AUC_PP_	0.176	0.453**	0.229	0.253	0.543**				
HDL-C	− 0.051	0.071	− 0.176	0.168	0.160	0.004			
LDL-C	0.204	0.286*	0.310*	− 0.110	0.066	0.262*	0.091		
TG	0.098	0.219	0.252	− 0.151	− 0.016	0.024	− 0.256*	0.273*	
d-ROMs	0.004	0.217	− 0.023	0.329*	0.448**	0.307*	0.044	− 0.098	0.072

*FPG* fasting plasma glucose, *MGL* mean glucose level over 24 h, *HbA1c* hemoglobin A1c, *%CV* percentage coefficient of variation for glucose, *MAGE* mean amplitude of glycemic excursions, *AUC*_*PP*_ area under the postprandial glucose curve, *HDL-C* high-density lipoprotein cholesterol, *LDL-C* low-density lipoprotein cholesterol, *TG* triglycerides, *d-ROMs* diacron-reactive oxygen metabolites

*p < 0.05, **p < 0.01

Table 3 shows the correlation between d-ROMS and markers of BP control. Significant correlations were observed between d-ROMs and nighttime SD of SBP (r = 0.274; p = 0.034), DBP (r = 0.262; p = 0.043), and CV of SBP (r = 0.266; p = 0.040). However, no significant correlation was observed between d-ROMs and other markers of BP variability.

**Table 3:** Correlations between d-ROMs and markers of BP control

	Daytime AV of SBP	Daytime SD of SBP	Daytime CV of SBP	Daytime AV of DBP	Daytime SD of DBP	Daytime CV of DBP	Nighttime AV of SBP	Nighttime SD of SBP	Nighttime CV of SBP	Nighttime AV of DBP	Nighttime SD of DBP	Nighttime CV of DBP
Daytime SD of SBP	0.163											
Daytime CV of SBP	− 0.190	0.932**										
Daytime AV of DBP	0.772**	0.028	− 0.242	–								
Daytime SD of DBP	0.282*	0.687**	0.572**	0.190								
Daytime CV of DBP	− 0.056	0.649**	0.658**	− 0.244	0.898**							
Nighttime AV of SBP	0.842**	0.061	− 0.219	0.710**	0.225	− 0.081						
Nighttime SD of SBP	0.224	0.273*	0.205	0.004	0.256*	0.277*	0.232					
Nighttime CV of SBP	− 0.155	0.241	0.305**	− 0.303*	0.163	0.321*	− 0.225	0.888**				
Nighttime AV of DBP	0.662**	0.000	− 0.213	0.859**	0.166	− 0.204	0.851**	0.054	− 0.322*			
Nighttime SD of DBP	0.148	0.216	0.177	0.121	0.307*	0.271*	0.139	0.608**	0.574**	0.167		
Nighttime CV of DBP	− 0.180	0.205	0.278	− 0.307*	0.207	0.364**	− 0.289*	0.542**	0.709**	− 0.340**	0.738**	
d-ROMs	0.026	− 0.029	− 0.033	− 0.040	0.030	0.078	0.012	0.274*	0.266*	− 0.028	0.262*	0.249

*AV* average, *SD* standard deviation, *CV* coefficient of variation, *SBP* systolic blood pressure, *DBP* diastolic blood pressure, *d-ROMs* diacron-reactive oxygen metabolites

*p < 0.05, **p < 0.01

Additional file 1: Table S1 shows the relationship between glucose variability, nighttime BP variability, glucose variability + nighttime BP variability, and oxidative stress. Oxidative stress was significantly higher in the both high glucose variability and BP variability group compared with both low glucose variability and BP variability groups.

In the univariate analysis, a strong correlation was observed between SDs of BP and CVs of BP (Table 3). Therefore, we designed two independent models: model 1 included SDs of BP and model 2 included CVs of BP. Multivariate analysis identified MAGE, eGFR, smoking, and nighttime SD and CV of DBP as independent and significant determinants of d-ROMs (model 1: adjusted multiple R^2^ = 0.396, model 2: adjusted multiple R^2^ = 0.439; Table 4).

**Table 4:** Linear multivariate analyses with changes in d-ROMs as dependent variables

	Dependent variables: d-ROMs (U.CARR)			
	β coefficient	t value	p value	Full-model R^2^
Model 1			< 0.001**	0.396
MAGE	0.311	2.831	0.006**	
eGFR	−0.228	−2.211	0.031*	
Smoking	0.365	3.376	0.001**	
Nighttime SD of DBP	0.225	2.036	0.047*	
Model 2			< 0.001**	0.439
MAGE	0.321	3.147	0.003**	
eGFR	− 0.204	− 2.032	0.047*	
Smoking	0.379	3.646	0.001**	
Nighttime CV of DBP	0.258	2.484	0.016*	

Multiple stepwise regression analysis, with d-ROMs as the dependent variable, adjusted for sex (female), age, duration of diabetes, body mass index, smoking status (current), use of insulin, glucose-like peptide-1 receptor agonists, dipeptidyl peptidase 4 inhibitors, sulfonylureas, glinides, α-glucosidase inhibitors, metformin, thiazolidine, sodium glucose cotransporter 2 inhibitors, statins, angiotensin II receptor blockers, calcium channel blockers, diuretics, α and β blockers, estimated glomerular filtration rate, FPG, MGL, HbA1c, %CV, MAGE, AUC_PP_, daytime AV of SBP, daytime SD of SBP, daytime AV of DBP, daytime SD of DBP, nighttime AV of SBP, nighttime SD of SBP, nighttime AV of DBP, nighttime SD of DBP, HDL-C, LDL-C, and TG

*d-ROMs* diacron-reactive oxygen metabolites, *FPG* fasting plasma glucose state, *MGL* mean glucose level over 24 h, *HbA1c* hemoglobin A1c, *MAGE* mean amplitude of glycemic excursions, *AUC*_*PP*_ total area under the postprandial plasma glucose curve, *AV* average, *SD* standard deviation, *CV* coefficient of variation, *SBP* systolic blood pressure, *DBP* diastolic blood pressure, *HDL-C* high-density lipoprotein cholesterol, *LDL-C* low-density lipoprotein cholesterol, *TG* triglyceride

*p < 0.05, **p < 0.01

### Relationship between glucose and BP variabilities as well as between LF/RF ratio and CVR–R

**Fig. 2:**
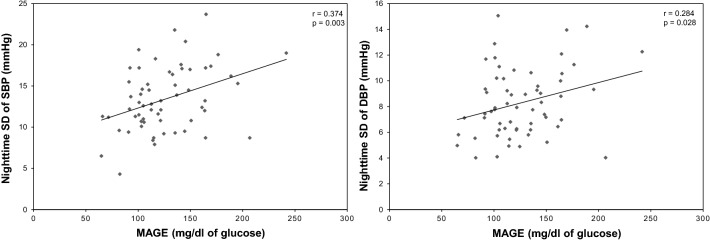
Correlations between mean amplitude of glycemic excursions (MAGE) and nighttime blood pressure variability

Additional file 2: Table S2 shows the correlation between MAGE and BP variability. Significant correlations were observed between MAGE and nighttime SD of SBP (r = 0.374; p = 0.003), DBP (r = 0.284; p = 0.028,) and nighttime CV of SBP (r = 0.276; p = 0.033; Fig. 2). However, no significant correlations were observed between MAGE and daytime AV of SBP and DBP, daytime SD and CV of SBP and DBP, and nighttime AV of SBP and DBP.

Additional file 3: Table S3 shows the correlation between LF/HF ratio and CVR–R as well as that between glucose variability and markers of BP variability. Significant correlation was observed between MAGE and LF/HF ratio at nighttime (r = 0.319; p = 0.013). However, no significant correlations of BP variability with LF/HF ratios at 24 h, daytime, and nighttime as well as with CVR–R were observed.

## Discussion

A previous study has demonstrated that in patients with T2DM without hypertension, oxidative stress is associated with glucose variability indexes, such as continuous overall net glycemic action, as well as with BP variability indexes, such as percent changes in systolic and DBP from daytime to nighttime [25]. However, no other study has investigated the relationship between oxidative stress and glucose and BP variability in patients with T2DM and hypertension. Therefore, the present study has been the first to demonstrate that oxidative stress is associated with not only daily glucose but also daily BP variability in patients with T2DM and hypertension. Our findings are significant and suggest the importance of not only HbA1c, maximum SBP, and DBP but also glucose and BP variability in the clinical management of T2DM with hypertension.

The present study showed an association between oxidative stress, eGFR, and smoking, a finding consistent with that presented in previous studies [26, 27]. With regard to glucose metabolism, previous clinical studies have shown that glucose variability, evaluated through CGM, was associated with oxidative stress [16], a result similar to that presented in the present study. Conversely, only a few studies have investigated the association between BP variability, evaluated through 24 h ABPM, and oxidative stress in hypertension. The present study showed that nighttime but not daytime BP variability was associated with oxidative stress in patients with T2DM and hypertension. In support of our findings, Eguchi et al. [28] demonstrated that nighttime BP variability was a strong predictor for cardiovascular disease in patients with T2DM and hypertension. Another study on patients with diabetes showed that parasympathetic nerve activity was relatively low during the night, resulting in nocturnal sympathetic predominance [29]. Therefore, the involvement of diabetic autonomic nerves is surmised to be able to explain the association between nighttime BP variability and oxidative stress. On the other hand, daytime BP variability was not related to oxidative stress, even though SDs of BP variability was higher during daytime than nighttime. The reason was due to the indicator that SDs of BP variability is dependent on average of BP. As a result, in the analysis using CVs of BP variability adjusted by average of BP, CVs of BP variability during nighttime associated with oxidative stress.

**Fig. 3:**
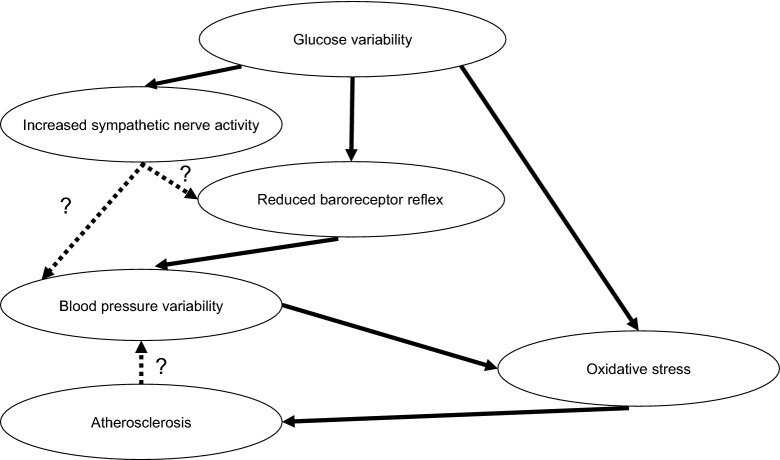
Hypothesis regarding the association between glucose and blood pressure variabilities

Reduced baroreceptor reflex, increased sympathetic nerve activity, and progress of atherosclerosis are factors contributing to BP variability [30, 31]. Further, patients with diabetes and hypertension have been reported to have greater BP variability compared with those without such conditions [32]. This is due to reduced baroreceptor reflex sensitivity [33], relatively increased sympathetic activity [34], and progress of atherosclerosis in T2DM [35]. Although baroreceptor reflex sensitivity has been associated with diabetic neuropathy [36], the present study found no relationship between BP variability, LF/HF ratio, and coefficient of variation in the R–R intervals. This result may support previous findings wherein baroreceptor reflex sensitivity had been found to be significantly reduced before the onset of autonomic dysfunction in patients with diabetes [37]. Conversely, one recent report showed that glucose variability is inversely associated with baroreceptor reflex sensitivity [38]. Our results show a correlation between glucose variability and nighttime BP variability, which supports the notion that glucose variability attenuates baroreceptor reflex sensitivity. In addition, our results show a correlation between glucose variability and LF/HF ratio at nighttime. Our findings showed that glucose variability was related to nighttime but not daytime BP variability. Sasaki et al. [39] reported that impaired glucose tolerance and diabetes mellitus are associated with BP variability. They suggested that glucose metabolism disorder may modulate BP variability through arteriosclerosis and decreased baroreceptor reflex. Thus, we present our hypothesis regarding the association between glucose and BP variabilities in Fig. 3. In addition, we investigated whether MAGE and BP variabilities are synergistic or additive to d-ROMs. There was no significant interaction between MAGE and nighttime SD of DBP. This finding indicates that MAGE and nighttime SD of DBP additively affect d-ROMs (two-away ANOVA; p = 0.497). However, we did not obtain consistent results regarding effects of sympathetic activities on the association between glucose and BP variabilities in the present study. Therefore, further investigation into the association of sympathetic nerve activity with glucose and BP variabilities is warranted.

Reports have shown a relationship between cardiovascular disease and BP variability evaluated through ABPM. Accordingly, several studies have determined nighttime SBP variability to be a risk factor for cardiovascular disease [28, 40, 41]. On the other hand, the present study showed that nighttime DBP variability was associated with oxidative stress. However, the mechanism leading to this result is unknown. The results of the ONTARGET study, wherein, regardless of SBP level, cardiovascular disease increased when DBP was low among patients with diabetes were reported [42]. This can be explained by the following pathophysiologic mechanism: low DBP could compromise blood flow to the target organs, impairing coronary perfusion and causing cardiac ischemia [43]. Therefore, we considered the possibility that hypotension caused by higher DBP variability causes oxidative stress. However, at the quartile of DBP variability, DBP was less than 60 mmHg at the same rate (first quartile 〈nighttime SD of DBP: 5.3 ± 0.8〉: 18.3%, second quartile 〈nighttime SD of DBP: 7.0 ± 0.5〉: 13.3%, third quartile 〈nighttime SD of DBP: 8.9 ± 0.6〉: 16.7%, fourth quartile 〈nighttime SD of DBP: 11.9 ± 1.5〉: 16.7%, p = 0.723).

The present study has several limitations worth noting. First, this was a cross-sectional study, precluding the evaluation of any cause–effect relationship between BP variability and oxidative stress. Further studies should examine whether interventions aimed at reducing BP variability are required. Second, given that the sample size was relatively small, any subgroup comparison may lack statistical power. Third, other markers of oxidative stress had not been measured for comparison. It is reported that the d-ROMs measured in the present study are not only quick and inexpensive for use in the clinical settings but also have high reproducibility and correlation with Electron Spin Resonance [44]. Actually, it has been reported that d-ROMs predict morbidity and mortality [45, 46]. We also reported their association with glucose variability in T2DM [16, 17]. However, d-ROMs are a new oxidative stress marker, and there are not as much data for them as for 8-hydroxydeoxyguanosine [14] or 8-iso-prostaglandin F2α [9, 15]. Fourth, because the glycemic control in these subjects was poor, hyperglycemia and glucose variability may have had a significant effect on the results of this study. In the future, we should perform a study, including patients with good glycemic control. Finally, BMI ≥ 25 is diagnosed as obesity in Japan [47]. As the average BMI of participants in the present study is 27.6, there is a possibility that obstructive sleep apnea (OSA) is included in some of the participants. OSA causes BP variability during sleep [48], and repetitive oxygen desaturation affects oxidative stress [49]. In the present study, it is possible that OSA may have influence on the results from nighttime BP variability related to oxidative stress.

## Conclusions

In conclusion, the present study has been the first to show that oxidative stress is associated with glucose and BP variability in patients with type 2 diabetes mellitus and hypertension. Although further intervention studies are required to determine whether reducing not only glucose variability but also BP variability is associated with reduced oxidative stress, the results presented herein suggest that glucose and BP variability is an important factor affecting oxidative stress.

## Additional files

**Additional file 1: Table S1.** Relationship between glucose variability, nighttime blood pressure variability, glucose variability + nighttime blood pressure variability, and oxidative stress.

**Additional file 2: Table S2.** Correlations between MAGE and blood pressure variability.

**Additional file 3.** Correlations between LF/HF ratio and CVR–R, glucose variability, and markers of blood pressure control.

## Abbreviations

T2DM: type 2 diabetes mellitus
BP: blood pressure
CGM: continuous glucose monitoring
ABPM: ambulatory blood pressure monitoring
d-ROMS: diacron-reactive oxygen metabolites
SBP: systolic blood pressure
DBP: diastolic blood pressure
eGFR: estimated glomerular filtration rate
FPG: fasting plasma glucose
LDL-C: low-density lipoprotein cholesterol
HDL-C: high-density lipoprotein cholesterol
TG: triglycerides
MGL: mean glucose level
%CV: percentage coefficient of variation for glucose
MAGE: mean amplitude of glycemic excursions
AUC_PP_: area under the postprandial plasma glucose curve
AV: average
LF/HF ratio: the ratio of low- to high-frequency power
CVR–R: coefficient of variation in the R–R intervals
