# Supplementary material for: Association of glucose and blood pressure variability on oxidative stress in patients with type 2 diabetes mellitus and hypertension: a cross-sectional study
Source: Diabetol Metab Syndr. 2019 Apr 11;11:29. doi: 10.1186/s13098-019-0425-y (PMC6460855; doi:10.1186/s13098-019-0425-y)
Supplement: Supplementary file 1 — Additional file 1: Table S1. Relationship between glucose variability, nighttime blood pressure variability, glucose variability + nighttime blood pressure variability, and oxidative stress. [file 13098_2019_425_MOESM1_ESM.docx]

**Additional file 1: Table S1** Relationship between glucose variability, night-time blood pressure variability, glucose variability + night-time blood pressure variability, and oxidative stress

|  | Group 1 | Group 2 | Group 3 | Group 4 | P |
| --- | --- | --- | --- | --- | --- |
| N | 17 | 13 | 13 | 17 |  |
| d-ROMs (U.CARR) | 318.4 ± 45.7 | 364.5±70.9 | 359.9±52.0 | 384.7±78.0* | 0.028 |
| FPG (mg/dl) | 121.9 ± 26.1 | 152.6±36.3 | 145.1±40.5 | 151.3±26.7 | 0.030 |
| MGL (mg/dl) | 158.7 ± 23.6 | 194.5±36.8* | 172.7±39.1 | 203.4±31.5** | 0.001 |
| HbA1c (%) | 7.9 ± 1.2 | 8.7 ± 0.9 | 8.8 ± 1.3 | 8.9 ± 1.2 | 0.059 |
| MAGE (mg/dl) | 95.2 ± 16.7 | 145.3 ± 23.2** | 103.2 ± 8.0^♯♯^ | 159.3 ± 30.6** | <0.001 |
| %CV | 20.5 ± 4.4 | 26.8 ± 6.3* | 20.9 ± 4.0^♯^ | 26.8 ± 4.9** | <0.001 |
| Night-time AV of SBP (mmHg) | 117.4 ± 12.5 | 125.6 ± 19.5 | 114.2 ± 13.4 | 130.5 ± 20.7 | 0.039 |
| Night-time SD of SBP (mmHg) | 10.2 ± 2.7 | 12.8 ± 3.9 | 14.8 ± 2.5** | 16.1 ± 3.6**^♯^ | <0.001 |
| Night-time CV of SBP (mmHg) | 8.7 ± 2.1 | 10.1 ± 2.3 | 13.1 ± 2.8**^♯^ | 12.6 ± 3.6** | <0.001 |
| Night-time AV of DBP (mmHg) | 66.9 ± 10.1 | 72.2 ± 11.1 | 67.3 ± 10.8 | 76.8 ± 15.6 | 0.081 |
| Night-time SD of DBP (mmHg) | 6.1 ± 1.2 | 6.2 ± 1.0 | 10.4 ± 2.0**^♯♯^ | 10.4 ± 1.9**^♯♯^ | <0.001 |
| Night-time CV of DBP (mmHg) | 9.2 ± 1.8 | 8.8 ± 1.8 | 16.1 ± 5.0**^♯♯^ | 14.0 ± 2.8**^♯♯^ | <0.001 |

Group 1: low MAGE + low Night-time SD of BP

Group 2: High MAGE + low Night-time SD of BP

Group 3: low MAGE + High Night-time SD of BP

Group 4: High MAGE + High Night-time SD of BP

*P < 0.05; comparison to group 1, **P < 0.01; comparison to group 1

^♯^P < 0.05; comparison to group 2, ^♯♯^P < 0.01; comparison to group 2

d-ROMs: diacron-reactive oxygen metabolites, FPG: Fasting plasma glucose, MGL: Mean glucose level of 24hr blood glucose levels by continuous glucose monitoring system, HbA1c: hemoglobin A1c, MAGE: Mean amplitude of glycemic excursions, %CV: percentage coefficient of variation for glucose

AV: average, SD: standard deviation, CV: coefficient of variation, SBP: systolic blood pressure, DBP: diastolic blood pressure
