# Supplementary material for: Association of glucose and blood pressure variability on oxidative stress in patients with type 2 diabetes mellitus and hypertension: a cross-sectional study
Source: Diabetol Metab Syndr. 2019 Apr 11;11:29. doi: 10.1186/s13098-019-0425-y (PMC6460855; doi:10.1186/s13098-019-0425-y)
Supplement: Supplementary file 2 — Additional file 2: Table S2. Correlations between MAGE and blood pressure variability. [file 13098_2019_425_MOESM2_ESM.docx]

**Additional file 2: Table S2** Correlations between MAGE and blood pressure variability

|  | Daytime AV of SBP | Daytime SD of SBP | Daytime CV of SBP | Daytime  AV of DBP | Daytime  SD of DBP | Daytime  CV of DBP | Nighttime AV of  SBP | Nighttime SD of  SBP | Nighttime CV of SBP | Nighttime AV of DBP | Nighttime SD of  DBP | Nighttime CV of DBP |
| --- | --- | --- | --- | --- | --- | --- | --- | --- | --- | --- | --- | --- |
| MAGE | 0.156 | 0.037 | -0.008 | 0.144 | 0.031 | -0.019 | 0.180 | 0.374** | 0.276* | 0.187 | 0.284* | 0.226 |

*p < 0.05, **p < 0.01

MAGE: mean amplitude of glycemic excursions,

AV: average, SD: standard deviation, CV: coefficient of variation, SBP: systolic blood pressure, DBP: diastolic blood pressure
