# Supplementary material for: Association of glucose and blood pressure variability on oxidative stress in patients with type 2 diabetes mellitus and hypertension: a cross-sectional study
Source: Diabetol Metab Syndr. 2019 Apr 11;11:29. doi: 10.1186/s13098-019-0425-y (PMC6460855; doi:10.1186/s13098-019-0425-y)
Supplement: Supplementary file 3 — Additional file 3. Correlations between LF/HF ratio and CVR–R, glucose variability, and markers of blood pressure control. [file 13098_2019_425_MOESM3_ESM.docx]

**Additional file 3: Table S3** Correlations between LF/HF ratio and CVRR and glucose variability and markers of blood pressure control

|  | Daytime AV of SBP | Daytime SD of SBP | Daytime CV of SBP | Daytime  AV of DBP | Daytime SD of DBP | Daytime CV of DBP | Nighttime AV of  SBP | Nighttime SD of  SBP | Nighttime CV of  SBP | Nighttime AV of  DBP | Nighttime SD of  DBP | Nighttime CV of  DBP | MAGE |
| --- | --- | --- | --- | --- | --- | --- | --- | --- | --- | --- | --- | --- | --- |
| LF/HF ratio  of 24 h | 0.185 | −0.033 | -0.085 | 0.140 | 0.036 | 0.009 | 0.181 | −0.061 | -0.123 | 0.167 | −0.056 | -0.138 | 0.241 |
| LF/HF ratio  of daytime | 0.234 | 0.006 | -0.035 | 0.185 | 0.061 | 0.014 | 0.206 | −0.031 | -0.093 | 0.178 | −0.067 | -0.162 | 0.170 |
| LF/HF ratio  of nighttime | 0.063 | −0.080 | -0.019 | 0.040 | −0.004 | 0.011 | 0.099 | −0.089 | -0.088 | 0.105 | −0.049 | -0.120 | 0.319* |
| CVR-R | −0.143 | 0.016 | -0.030 | −0.058 | −0.147 | -0.083 | −0.167 | 0.029 | 0.097 | −0.094 | 0.062 | 0.147 | −0.006 |

*p < 0.05

LF/HF: low frequency power/high-frequency power,

CVR-R: coefficient of variation in the R-R intervals,

AV: average, SD: standard deviation, CV: coefficient of variation,

SBP: systolic blood pressure, DBP: diastolic blood pressure

MAGE: mean amplitude of glycemic excursions
